# Prevalence of chlamydia trachomatis infection among reproductive age women in sub Saharan Africa: a systematic review and meta-analysis

**DOI:** 10.1186/s12879-018-3477-y

**Published:** 2018-11-26

**Authors:** Siraj Hussen, Demelash Wachamo, Zemenu Yohannes, Endale Tadesse

**Affiliations:** 1 Department of Medical Laboratory Science, College of Medicine and Health Sciences, Hawassa University, Hawassa, Ethiopia; 2 Department of Public Health, Hawassa College of Health Sciences, South Nations and Nationalities Peoples’ Region, Hawassa, Ethiopia; 3 Department of Midwifery, College of Medicine and Health Sciences, Hawassa University, Hawassa, Ethiopia

**Keywords:** Systematic review, Meta-analysis, *Chlamydia trachomatis*, Reproductive age women, Sub-Saharan Africa

## Abstract

**Background:**

*Chlamydia trachomatis* is the most common curable sexual transmitted bacterial infection in the world, including Sub-Saharan Africa. There is nil systematic review and meta-analysis on *Chlamydia trachomatis* infection in Sub-Saharan Africa among reproductive age women. Therefore, this study was carried out to determine the pooled prevalence of *chlamydia trachomatis* infection in Sub-Saharan Africa among reproductive age women.

**Methods:**

A comprehensive literature search was conducted from biomedical data bases: Medline, PubMed, EMBASE, Google scholar, HINARI and Cochrane Library using a special index search terms (medical subject headings (MeSH), title and abstract. The Cochrane Q test and I^2^ statistics was used to test heterogeneity and publication bias was assessed using Begg’s and Egger’s tests. Results were presented in tables, figures and funnel plot. Data were pooled in a meta-analysis using a random effects model.

**Results:**

Twenty-four studies were included in this meta–analysis. There was a high level of heterogeneity among studies. The pooled prevalence of *Chlamydia trachomatis* infection in Sub-Saharan Africa among reproductive age women *was* 7.8% (95% CI: 5.6–10.6).

**Conclusion:**

This review showed that *Chlamydia trachomatis* infection is high in Sub-Saharan Africa among reproductive age group women. This evidence suggests that governmental and non-governmental organization shall give attention for primary prevention of this infection. Likewise, in resource limited countries policy makers, stakeholders and health care providers’ due attention for *Chlamydia trachomatis* specific and rapid diagnostic test, treatment in any medical out and in patient clinics for reproductive age women.

## Background

*Chlamydia trachomatis* is the major public health concern across the globe,and the main cause of sexual transmitted infections throughout the world, especially Sub-Saharan Africa [1]. The World Health Organization (WHO) estimated that 50 million women were newly infected with *Chlamydia trachomatis* worldwide, of which 34 million were in Sub-Saharan Africa and South/Southeast Asia.It is the most implicated organism that causes infertility and pelvic inflammatory disease [2–5].

*Chlamydia trachomatis* is the most common curable sexual transmitted bacterial infection in the world, with an estimated 4–5 million new cases each year [6]. WHO estimated that, the incidence of *Chlamydia trachomatis* is high in sub-Saharan Africa, which is more than 10 million new infection annually [2].

*Chlamydial* infection in women is commonly asymptomatic. Undetected and untreated *Chlamydial* infection can ascend upper genitalia that may cause pelvic inflammatory disease (PID), infertility, ectopic pregnancy and chronic pelvic pain [7, 8]. *Chlamydial* infection in women show that different clinical manifestations and associated disease like: cervicitis,endometritis, salpingitis, pelvic inflammatory disease, infertility, preterm rupture of membranes, perihepatitis, while most of women do not get medical care, because more than three forth of women are commonly asymptomatic [9].Untreated Chlamydial infection cause up to 40% of pelvic inflammatory disease cases, one in four of these will result in infertility [10].

Untreated genital infection in sub-Saharan Africa can cause up to 85% of infertility among women who seek infertility treatment and care. Undetected and untreated Chlamydial infections during pregnancy can increase risk of cervicitis, endometritis, salpingitis, pelvic inflammatory disease, infertility, perihepatitis, premature rupture of the membranes, low birth weight,chorio amnionitis, neonatal sepsis and conjunctivitis in new born [11, 12]. Whereas, the risk of developing PID after lower genital tract chlamydial infection varies considerably, up to 30%, and the risk of developing tubal infertility after PID is 10–20% [7].

Chlamydial infection can occur at any anatomical site of sexual contact including endocervix, urethra, rectum, and oropharynx, which causes pelvic inflammatory disease, infertility, ectopic pregnancy and chronic pelvic pain for women [13].

Throughout our search and knowledge, there is no systematic review and meta-analysis regarding *Chlamydia trachomatis* infection among reproductive age women in Sub-Saharan Africa. This study is used as an input for clinician, public health experts and stake holders for possible interventions.

## Methods

### Study design and search strategy

A systematic review and meta-analysis was done using published articles on prevalence of *Chlamydia trachomatis* in Sub-Saharan Africa. A comprehensive literature search was conducted from biomedical data bases: Medline, PubMed, EMBASE, Google scholar, HINARI and Cochrane Library using a special index search terms (medical subject headings (MeSH) “prevalence of *Chlamydia trachomatis AND* Sub-Saharan Africa, *Chlamydia trachomatis* AND reproductive age group, *Chlamydia trachomatis* OR Neisseria gonorrhea, *Chlamydia trachomatis* OR sexual transmitted infection”, title and abstract. The limit of language was English and the limit of study group was human. Searching of articles were carried out from March to October 01, 2017.

### Study selection and data extraction

Cross-sectional studies published in English language from 1997 to 2017 were included. Articles that assessed prevalence of *Chlamydia trachomatis* infection among reproductive age group who attended ANC, family planning clinic, STI clinic, Gynecology clinic and in general population were used. Age restriction was imposed. Reproductive age group women were defined as those of age 15–49 years.

The critical appraisal was done before the extraction of data. Data extraction was carried out using the Downs and Black checklist [14]. All essential information was extracted from the final selected studies. It contains study year, population characteristics, sample size, prevalence, age, and Chlamydia *trachomatis* screening technique. Four authors independently reviewed the studies and inconsistencies were resolved through discussion and consensus.

### Quality assessment

The quality of selected articles were assessed using 12 point scoring system based on Downs and Black check lists. These are: (clarity of objective, reported response rate which scored ≥80%, clear data collection methods and procedures, study design clearly described, sample representativeness of the entire population, the main finding of the study clearly described, suitable sampling methods, reliable measurement of outcome variable, use of appropriate statistical analysis method, and quality assurance methods). Mean quality score was used to assess the quality of included studies in the meta-analysis. Studies which scored above the mean of the quality score were grouped into the high-quality score, and those below the mean were grouped as low-quality score and not include in the meta-analysis [14].

### Statistical analysis

Data entry and analysis were done using Comprehensive Meta-Analysis (version 3.1).The pooled prevalence of *Chlamydia trachomatis* with 95%CI was obtained using the random effects model, due to the possibility of heterogeneity among the studies.

### Sub-group analysis

Sub-group analysis was conducted based on type of study population; (Community based, FCSWS Health facility based), Geographical zone; (East Africa, Middle Africa, Southern Africa and West Africa), laboratory diagnostic methods (ICT and PCR) and Year of study; (1997–2001, 2002–2006, 2007–2011, and 2012–2016).

### Heterogeneity and publication bias

The heterogeneity of studies were assessed using Cochran’s Q test and I^2^ test statistics. A Cochran’s Q test *P* < 0.10 is indicated that heterogeneity between the studies [15]. The level of I^2^ test statistics of 25, 50 and 75% are used low, medium and high heterogeneity, respectively [16]. Publication bias was assessed by Egger’s and Begg’s test, and *p*-value less than 0.05 is statistically significance, and there is publication bias [17].

## Results

### Identified studies

**Fig. 1:**
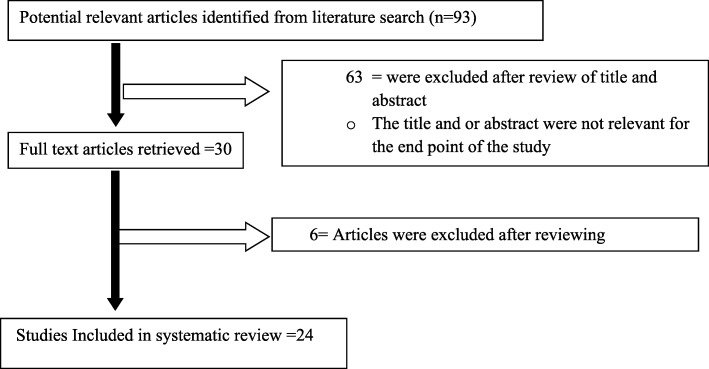
Flow diagram of studies reviewed, screened and included

A total of 93 records were retrieved through electronic database searching. Records were screened using their titles, abstracts and through full article review. Accordingly, a total of 63 articles were excluded using their title and abstract review. Thirty articles were assessed for eligibility and six article was excluded by exclusion criteria in the study. Finally, 24 articles were included in this meta-analysis (Fig. 1). The Cohran’s Q (905.3) and I^2^ statistics (I^2^ = 97.459%; *p* < 0.0001) revealed that high heterogeneity among studies. However, neither Egger’s test (*p* = 0.231) nor Begg’s test (*p* = 0.085) gave evidence of publication bias, which indicate to use random effects model.

### Study characteristics

The total study population size screened for *Chlamydia trachomatis* and involved in this systematic review and meta-analysis were 17,119.Among these, 9606 were screened at community based studies [18–23], about 2638 were FCSWS [19, 20, 24–28] and 4875 were at health facility based studies [28–38]. The sample size of study population varied from 100 [24] to 4886 [19], and were conducted between the year 1997–2001 [19, 22, 30], 2002–2006 [25], 2007–2011 [21, 26, 31] and 2012–2016 [23, 27, 34–36]. Geographically,the population screened for *Chlamydia trachomatis* four regions of Sub-Saharan Africa: East Africa [19, 22, 27, 32–35, 37],West Africa [18, 20–24, 26, 28–30], Southern Africa [22, 31, 39], and middle Africa [25, 38] (Table 1).

**Table 1:** *Chlamydia trachomatis infection* among reproductive age women in different study populations in different regions of Sub Saharan Africa from 1997 to 2016 [18–34]

Authors, publication year [Ref]	Country	Study population	Sample size	Prvalence(%)	Specimen	Age group	Diagnostic methods
Yirenya et al., 2014 [18]	Ghana	Community	191	6.3	Endocervical swabs	15–49	PCR
Obasi et al., 2001 [19]	Tanzania	Community	4686	2.4	Urine	15–19	PCR
Wariso et al., 2012 [20]	Nigeria	Student	400	11	Urine	16–30	PCR
Ikeme et al., 2011 [21]	Nigeria	Community	286	29.4	Blood	20–34	ICT
Buve et al., 2001 [22]	Benin	Community	962	1.3	Urine	15–49	*PCR*
Buve et al., 2001 [22]	Cameron	Community	1016	9.4	Urine	15–49	*PCR*
Buve et al., 2001 [22]	Kenya	Community	821	4.5	Urine	15–49	*PCR*
Buve et al., 2001 [22]	Zambia	Community	890	2.9	Urine	15–49	PCR
Arize et al., 2014 [23]	Nigeria	Students	354	30.2	Endocervical swabs	15–30	ICT
Abubakari et al.,2016 [24]	Ghana	FCSWs	100	19	Endocervical swabs	18–35	ICT
Vandepitte et al.,2007 [25]	Congo	FCSWs	502	8.4	Vaginal swabs	15–49	PCR
Opoku & Sarkodie,2014 [26]	Ghana	FCSWs	1070	4.8	vaginal swabs	18–35	*ICT*
Francis et al.*,* 2014 [27]	Tanzania	FCSWs	966	12	Blood (Serum)	18–44	PCR
Apea-Kubi,2014 [28]	Ghana	OB and Gyn	465	3	Endocervical swabs	15–49	PCR
Gomes et al., 2001 [29]	Guinea-Bissau	STI and FP	200	4	Endocervical swabs	15–49	PCR
Luján et al., 2008 [30]	Mozambique	ANC	1119	4.1	urine	15–49	PCR
Kohli et al., 2013 [31]	Kenya	OPD	300	6	Vaginal swabs	18–45	ICT
Adesiji et al., 2015 [32]	Nigeria	FP and Gyn	140	0.7	Endocervical swabs	15–49	ICT
Tadesse et al., 2016 [33]	Hawassa	FP and Gyn	322	18.9	Endocervical swabs	15–49	ICT
Musa et al.*,* 2016 [34]	Uganda	Gyn	324	26.5	Endocervical swabs	15–49	ICT
Mainaet al.*,* 2016 [35]	Kenya	FP	261	13	Endocervical swabs	18–49	PCR
Peters et al.*,* 2014 [36]	South Africa	ANC	603	16	Vaginal swabs	18–49	PCR
Mayaud *et al.,* 2016 [37]	Tanzania	ANC	660	5.9	Endocervical swabs	15–49	ICT
Blankhart*et al*., 1999 [38]	C.A Rep.	ANC	481	6.2	Endocervical swab	15–49	PCR

*ANC* antenatal care, *OB* obstetrics, *FCSHS* Female commercial sex workers, *FP* Family planning, *Gyn* gynecology, Community based study (all reproductive age women who live in the study area), *ICT* Immuno chromatographic test and *PCR* Polymerase chain reaction

### Meta-analysis

**Fig. 2:**
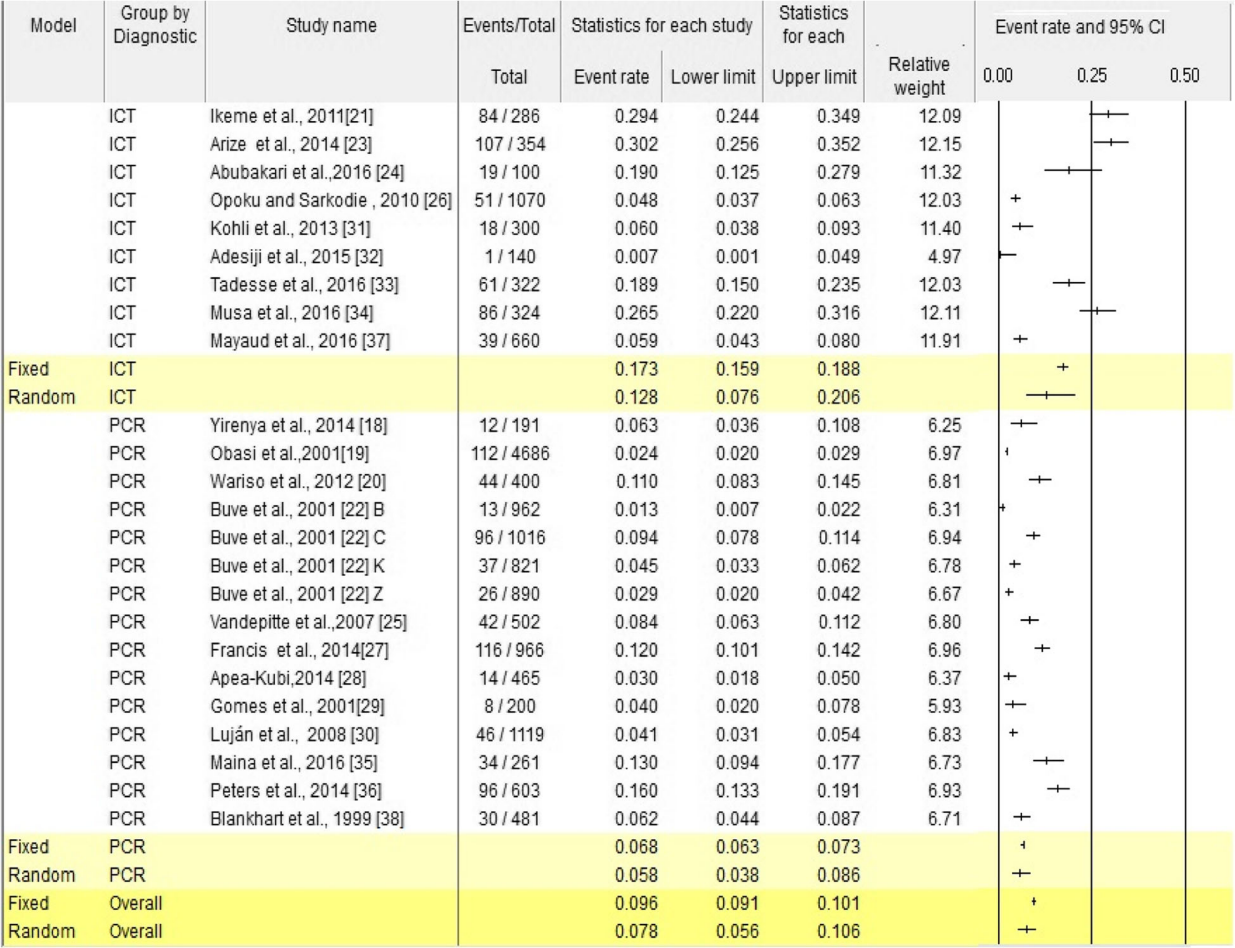
The meta-analysis and forest plot presentation of *C.trachomatis* prevalence from 1997 to 2016 (Citations of studies used in the analysis from top to bottom [18–37, 50]

**Fig. 3:**
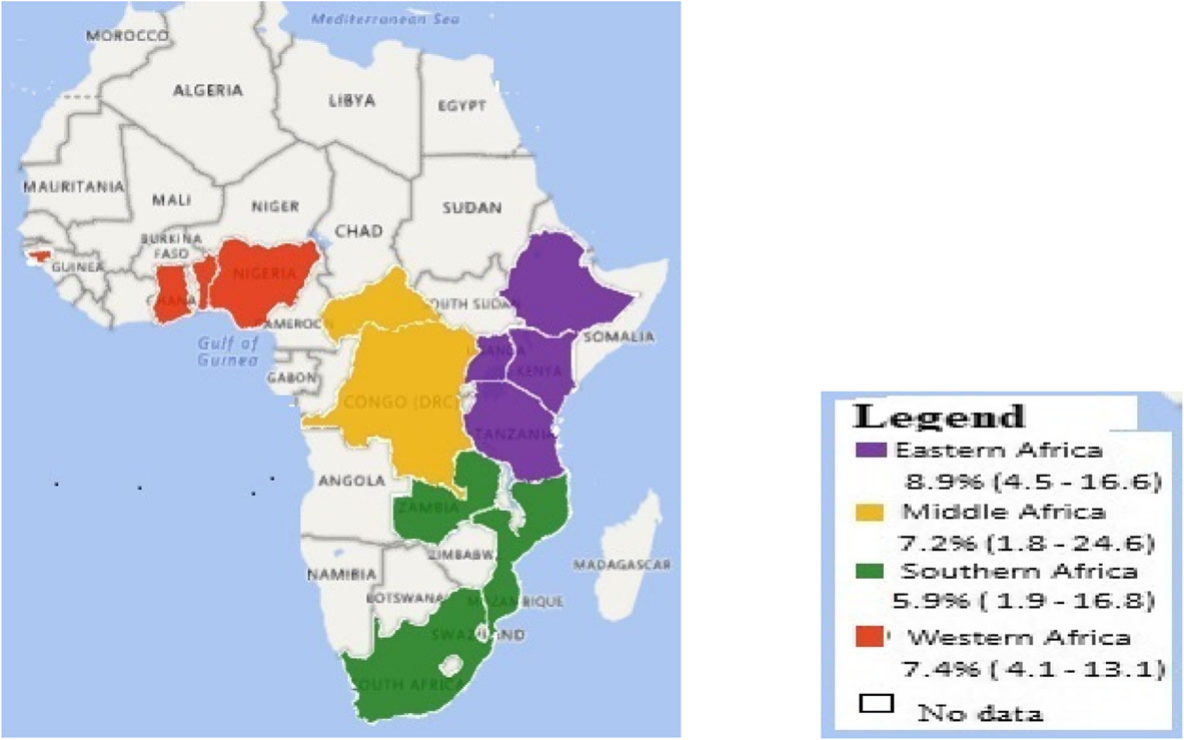
Prevalence of *Chlamydia trachomatis* in different regions of Sub-Saharan Africa among reproductive age group women, 1997–2016

The analysis of 24 studies, according to the Der Simonian-Laird random-effects model. The pooled prevalence of *C. trachomatis* among Sub-Saharan African reproductive age women was 7.8% (95% CI: 5.6–10.6) (Fig. 2). In particular, the pooled prevalence among subgroup was 9.7% (95% CI: 5.8–16.0) in FCSWs, 7.0% (95% CI; 3.2–14.7) in community based studies, and 7.6% (95% CI; 4.7–12.3) in health facility studies. Regarding year of study, 3.8% (95% CI; 2.1–6.7) from 1997 to 2001, 8.4% (95% CI; 1.8–31.1) from 2002 to 2006, 8.8% (95% CI; 3.7–19.5) from 2007 to 2011 and11.0% (95% CI; 7.3–16.4) from 2012 to 2016, while among diagnostic method 12.8% (95% CI; 7.6–20.6) screened by ICT, and 5.8% (95% CI; 3.8–8.6) screened by PCR (Table 2). Further, subgroup analysis was done among geographical location, 8.9% (95% CI; 4.5–16.6) in East Africa, 7.2% (95% CI; 1.8–24.6) Middle Africa, 5.9% (95% CI; 1.9–16.8) Southern Africa, and 7.4% (95% CI; 4.1–13.1) in West Africa (Fig. 3).

**Table 2:** Subgroup meta-analysis of C. trachomatis prevalence estimation in Sub Saharan Africa from 1997 to 2016

	Study parameters	Subgroup	Studies included	Prevalence %(95% CI)	I^2^%	*P*-v
*C. trachomatis*	study population	Community based	9	7.0(3.2–14.7)	98.678	< 0.0001
		CFSWs	4	9.7(5.8–16.0)	93.168	< 0.0001
		Health facility based	11	7.6(4.7–12.3)	95.763	< 0.0001
	Study year	1997–2001	7	3.8(12.1–6.7)	95.289	< 0.0001
		200–2006	1	8.4(1.8–31.1)	0.000	1.000
		2007–2011	3	8.8(3.7–19.5)	98.852	< 0.0001
		2012–2016	13	11.0(7.3–16.4)	94.753	< 0.0001
	Geographical zone	Eastern	8	8.9(4.5–16.6)	98.240	< 0.0001
		Middle	2	7.2(1.8–24.6)	42.502	0.187
		Southern	3	5.9(1.9–16.8)	98.008	< 0.0001
		Western	11	7.4(4.1–13.1)	97.181	< 0.0001
	Diagnostic method	ICT	9	12.8(7.6–20.6)	97.083	< 0.0001
		PCR	15	5.8(3.8–8.6)	96.230	< 0.0001

## Discussion

*Chlamydia trachomatis* is an important public health problem across the globe, including Sub-Saharan Africa. Most developed countries have implemented specific chlamydial infection control programs that vary from case management to opportunistic screening of high risk groups and annual screening program for sexually active women age < 25 years to tackle the problem. These countries decreased chlamydial infection and its complication, while in developing countries the management is still syndromic approach, and its infection and complications are still huge burden in Sub-Saharan Africa [40], because of its asymptomatic nature of the infection in most patients left unnoticed and remain untreated for longer period of time, there by transmitting the infection to their sexual partner(s). Annual screening of *Chlamydia trachomatis* in low income countries in all sexually active women aged < 25 years isn’t applied, whereas after complications the cost of diagnosis and treatment is high, which is compared to annual screening [7, 11]. In resource limited countries, reports of *Chlamydia trachomatis* represents only ‘tip of ice berg’, most of women have asymptomatic stage [11].

Based on the available data, the present study attempted to synthesize prevalence of *chlamydia trachomatis* in Sub-Saharan Africa among reproductive age women. In most studies the prevalence of *chlamydia trachomatis* is widely different from time to time, region to region, study population, study setting and type of laboratory diagnosis method.

This systematic review and meta-analysis showed that *Chlamydia trachomatis* among reproductive age group women in Sub-Saharan Africa was 7.8%, among diagnostic method 5.8% screened by PCR and 12.8% screened by ICT. This finding is inconsistent with WHO 2008 estimated in Africa is 2.6% [11], in 2005 is 4% [2] and global estimated is 4.2% [6].This finding is in line with systematic review in women attending antenatal care estimated prevalence of 6.9%, and the highest prevalence is predominantly at younger age < 25 years for chlamydial infection [41, 42].

In this study, the prevalence in east Africa was 8.9%. This finding is in agreement with the 6.9% reported in a systematic review and meta-analysis in East/Southern Africa, and 6.1% (95% CI: 4.0–8.3) in West/Central Africa. But, lower than a single counties reviewed studies like 4.9–14% in China, 0.1–35.9% in India, 5.7–16.2% in Thailand, 19.3% in Mongolia, and 41–44% in Bangladesh [1]. The difference might be, in this study, most of studies takes place in health facilities and around urban area, whereas studies in Asia is nationwide and screening strategy and diagnostic method quite different from Sub-Saharan Africa.

This finding is slightly higher than over all prevalence of a systematic reviewed in Australia is 4.6%, but with similar prevalence of 5.6% among adolescent and young adults [43] and in Europe, the prevalence ranged from1.7 to 17% depending on the setting, context and country [44] and this finding also slightly higher than over all prevalence in USA is 5% [45].

Over all prevalence in Australia is slightly lower than this study might be Australia women are more educated and treated at asymptomatic stage, because in Australia there is annual chlamydial infection screening for sexual active women age < 25 years.

This finding is slightly lower than with a systematic review in prison is 12.31% (95% CI:10.61, 14.01) for chlamydial infection in women, and a systematic review and meta-analysis in Iran, the pooled prevalence of the bacterium in the female population was 12.3% (95% CI: 10.6–14.2%) [46, 47]. The difference might be sociocultural, socioeconomically, screening strategy and types of laboratory diagnostic methods.

Pooled prevalence of *Chlamydia trachomatis infection* among commercial sex workers sub group was 9.7% (95% CI: 5.8–16.0)*.* This study is unlikely with the population based meta-analysis study conducted in Australia, for women age < 25 years reported 5.0% (95% CI: 3.1, 6.9), among women aged < 25 years attending sexual health, family planning or youth clinics, estimated prevalence was 6.2% (95% CI:5.1, 7.4; 10), and other key finding include pooled prevalence estimates of 22.1% (95% CI: 19.0, 25.3) for indigenous women < 25 years [48].

Potential limitations of this study, due to the nature of infection, most women are asymptomatic, or treated at private or traditional, self-treated and unreported or under reported, whereas *Chlamydia trachomatis is* under estimated. Another important limitation is that different diagnostic methods were used in the studies included in meta-analysis. The current estimates are limited to urogenital infections. But, chlamydial infection can be rectal and oropharyngeal infection. An important limitation is the use of reproductive age women as search term. Other limitations, among further others are the heterogeneity of data and lack of reproductive tract impact data.

Implication of this study; this review generate information on prevalence *of Chlamydia trachomatis* infection among reproductive age women in Sub-Saharan Africa. Therefore, Sub-Saharan Africa countries and their stakeholders use this information for evidence-based intervention, to establish rapid diagnostic test and to improve their national surveillance system of *Chlamydia trachomatis* infection. This systematic and meta-analysis is an input for developing countries, stakeholders and policy makers to develop diagnostic and treatment programs for *Chlamydia trachomatis* infections*. Chlamydia trachomatis* is a serious public health problem in developing countries, especially Sub Saharan Africa. STI including *Chlamydia trachomatis* over shadowed by HIV/AIDS and given less attention [20].

## Conclusion

This study revealed that *chlamydia trachomatis* infection in Sub-Saharan Africa among reproductive age group women is high. This evidence suggests that the government and non-government organization shall give attention for primary prevention of this infection. Likewise, in resource limited countries policy makers, stakeholders and health care providers’ due attention on *Chlamydia trachomatis* specific and rapid diagnostic test, treatment in any medical out and in patient clinics for reproductive age women.

## Abbreviations

ANC: Antenatal care clinic
C.T: *Chlamydia trachomatis*
FCSWs: Female commercial sex workers
FP: Family planning
ICT: Immunochromatographic test
OB: Obstetrics
PCR: Polymerase chain reaction
PID: Pelvic inflammatory disease
STI: Sexual transmitted infection
WHO: World health organization
